# 91074 Identification of monoclonal antibodies with broad reactivity against the malaria parasite variant surface antigen responsible for severe malaria

**DOI:** 10.1017/cts.2021.451

**Published:** 2021-03-30

**Authors:** Raphael Reyes, Louise Turner, Isaac Ssewanyana, John Rek, Bryan Greenhouse, Sebastiaan Bol, Thomas Lavstsen, Evelien M. Bunnik

**Affiliations:** 1 Department of Microbiology, Immunology and Molecular Genetics, The University of Texas Health Science Center, San Antonio, TX, USA; 2 Faculty of Health and Medical Sciences, University of Copenhagen, Copenhagen, Denmark; 3 Infectious Disease Research Collaboration, Kampala, Uganda; 4 Department of Medicine, University of California San Francisco, San Francisco, CA, USA

## Abstract

ABSTRACT IMPACT: This study aims to provide insight into naturally acquired immunity against severe malaria, thereby laying the foundation for the design of novel vaccine candidates to prevent severe disease as well as monoclonal antibody therapies to treat severe malaria. OBJECTIVES/GOALS: Severe malaria is caused by parasite surface antigens that contain high sequence diversity. Nevertheless, P. falciparum-exposed individuals develop antibody responses against these antigens. Our goal is to isolate antibodies with broad reactivity to understand how disease protection is acquired. METHODS/STUDY POPULATION: Our study cohort consists of Ugandan adults living in a malaria-endemic region with high transmission intensity, who are protected against severe malaria. Using fluorescently labeled probes of parasite surface antigens, we have isolated antigen-specific B cells from these donors. We then expressed the corresponding monoclonal antibodies in vitro. These antibodies were screened against a library of variant surface antigens to determine antibody breadth and potential to inhibit interaction of the parasite surface antigen with host receptors, a critical step in pathogenesis. Additionally, using a panel of variant surface antigen mutants, we have predicted the epitopes targeted by the broadest monoclonal antibodies. RESULTS/ANTICIPATED RESULTS: We have identified three monoclonal antibodies with exceptionally broad reactivity and inhibitory activity against our panel of severe disease-inducing variant surface antigens. We have identified two major sites targeted by these broadly reactive antibodies. The first site was associated with the largest breadth, but limited inhibitory potential, while the second site showed high-affinity antibody binding and inhibition of receptor binding. Interestingly, two of these three antibodies were very similar in structure, even though they were isolated from different donors. Isolation of antigen-specific B cells from additional donors will enable us to identify how common such broadly reactive antibodies are and allow the identification of additional epitopes DISCUSSION/SIGNIFICANCE OF FINDINGS: This study is the first to isolate broadly reactive antibodies that are likely to protect against severe malaria in naturally immune individuals. Further characterization of antibody-antigen interactions will inform the development of this surface antigen as a vaccine candidate for malaria.

